# Antiviral Effects and Mechanisms of Active Ingredients in Tea

**DOI:** 10.3390/molecules29215218

**Published:** 2024-11-04

**Authors:** Xinghai Zhang, Haonan Yu, Panjie Sun, Mengxin Huang, Bo Li

**Affiliations:** 1 Modern Service Industry Research Institute, Zhejiang Shuren University, Hangzhou, 310015, China; 601361@zjsru.edu.cn; 2 Department of Tea Science, Zhejiang University, Hangzhou 310058, China; 12416094@zju.edu.cn (H.Y.); 22316117@zju.edu.cn (P.S.); 22316137@zju.edu.cn (M.H.)

**Keywords:** tea, functional components, antiviral activity, mechanisms of antiviral activity

## Abstract

Viruses play a significant role in human health, as they can cause a wide range of diseases, from mild illnesses to severe and life-threatening conditions. Cellular and animal experiments have demonstrated that the functional components in tea, such as catechins, theaflavins, theanine, and caffeine, exhibit significant inhibitory effects on a diverse array of viruses, including influenza, rotavirus, hepatitis, HPV, and additional types. The inhibition mechanisms may involve blocking virus–host recognition, interfering with viral replication, enhancing host immune responses, and inhibiting viral enzyme activity. This article reviews the research progress on the antiviral effects of tea’s functional components and their related mechanisms, hoping to contribute to future studies in this field.

## 1. Introduction

Tea, a worldwide beverage, was derived in China and boasts a rich cultural influence over thousands of years. Its popularity is not solely due to its pleasant aroma and taste but also to its well-documented health benefits [1,2]. The bioactive components of tea, including catechins (phenolic compounds), flavonoids, theaflavins (polyphenol derivatives), theanine (an amino acid), and caffeine (an alkaloid), have attracted significant attention from scientists due to their potential benefits. These constituents which are mentioned above may play complementary roles in disease prevention and treatment [3,4]. Recent studies have increasingly focused on the antiviral capabilities of these components, especially in light of global viral infections and the search for effective, safe, and accessible antiviral solutions.

Viral infections pose serious public security problems, affecting the lives of millions of people and also leading to social unrest. The continuous mutation and adaptation of the virus poses a challenge to medical strategies. The mechanisms of viral infection are complex, involving the entry of viruses into host cells, replication, and the resulting immune response, which can lead to severe or chronic health conditions [5]. Although antiviral drugs and vaccines have been used to combat viral diseases for many years, they come with various limitations. For instance, with the development of drug resistance, the abuse and over-use of medicine is becoming more and more critical which may reduce the effectiveness of existing treatments [6]. In addition, the side effects of antiviral medications, including toxicity and adverse reactions, make their use restrictive, especially in adolescents and the older population. The high cost and the long development cycle of new antiviral drugs further complicate global efforts to address viral outbreaks. Thus, a novel antiviral agent has gained momentum with fewer side effects, a lower risk of resistance, and a wide spectrum of activity against different viruses.

Natural compounds, especially plant-derived active ingredients, have long been utilized in traditional Chinese medicine and are validated for their antiviral potential in contemporary research [7,8,9]. Among these natural antiviral agents, tea components have shown significant antiviral ability. Tea functional components can effectively interfere with the replication of viruses, including influenza, HIV, HSV, and hepatitis. The antiviral activities of tea are primarily attributed to its rich content of polyphenols, particularly catechins and flavonoids, which can reverse viral replication at multiple points within the viral life cycle. For example, epigallocatechin gallate (EGCG) has been found to influence the dynamic connection involving receptors located on host cells and viral proteins to prevent viral entry [10]. Additionally, viral RNA and DNA synthesis in viruses have been interfered with through the stage of viral replication [11,12]. The mechanisms by which tea components exert antiviral effects are complicated and confusing, often involving the adjustment of antioxidant activity, immune responses, and recognition between virus and host cells. These research results make tea an intriguing candidate for new antiviral treatments [13,14,15]. Consequently, tea functional components represent a potential option for antiviral therapies and for solving the limitations of conventional antiviral agents. This paper reviews the antiviral effects and mechanisms of tea components such as EGCG, theaflavins, theanine, and caffeine, offering new insights for the application of tea in the development of antiviral products.

## 2. Inhibitory Effects of Tea Functional Components on Different Viruses

### 2.1. Influenza Virus

The influenza virus, an RNA virus causing acute respiratory infectious diseases, infects humans, pigs, horses, and other organisms [16]. Unlike animal influenza viruses, human influenza viruses are classified into three categories, A, B, and C, according to the antigenicity of their nucleoprotein [17]. Furthermore, influenza viruses are categorized into subtypes determined by antigenic features of hemagglutinin (HA) and neuraminidase (NA). The primary variation in influenza viruses is antigenic, characterized by mutations in HA and NA surface antigens. Limited variations, known as antigenic drift, result in quantitative changes and restricted epidemic ranges. Conversely, extensive variations, referred to as antigenic shift, form new subtypes often causing widespread epidemics [18,19]. The viral nucleic acid comprises eight gene segments encoding hemagglutinin (HA), neuraminidase (NA), nucleoprotein (NP), matrix protein (M), polymerase (PB1, PB2, PA), and non-structural protein (NS) [20]. *HA* and *NA* genes are prone to mutations, resulting in changes in the encoded protein amino acid sequences, enabling the virus to escape detection from the immune defenses of the host. Changes in pathogenicity and propagation may arise from amino acid replacements in the receptor-binding domain of HA. Infected hosts experience inflammation and systemic reactions such as fever and weakness. Initially, the virus attaches to the host cell, with the HA protein recognizing N-acetylneuraminic acid on the cell membrane. After recognition, the virus is transported to the perinuclear cisternae, where the viral nucleic acid is exposed. Ultimately, the viral genome, once uncoated, is moved to the nucleus, where the processes of replication, assembly, and budding take place. Neuraminidase (NA) facilitates the enzymatic breakdown of viral receptors, enabling the release of new virus particles [21,22,23,24]. Tea and its bioactive components, such as catechins (e.g., EGCG), theaflavins, theanine, and caffeine, exhibit significant antiviral properties, particularly against influenza viruses [25]. These compounds can inhibit viral replication, disrupt viral envelope integrity, and modulate immune responses. Catechins, for example, block the attachment and entry of viruses into host cells, while theaflavins show synergistic effects when combined with other antiviral agents. Theanine enhances immune function by promoting the production of cytokines, and caffeine provides additional support by modulating inflammation [26,27,28,29,30]. Overall, tea compounds represent promising natural agents for both the prevention and complementary treatment of influenza.

#### 2.1.1. Hemagglutinin (HA)

Hemagglutinin (HA) is a glycoprotein spike that binds to sialic acid on target cells, promoting the merging between the viral envelope and the host cell membrane, thereby allowing the virus to enter the cell via endocytosis and initiate replication [25]. Nakayama et al. found that both EGCG and TF3, with EC_50_ values of 18–26 and 50–65 μM, could bind to HA and block its adhesion to MDCK cells, inhibiting the agglutination reaction and reversing virus transmission [26]. Further research by Imanishi et al. showed that tea extracts and polyphenolic compounds prevented the adsorption of influenza virus. It has been reported that tea extracts can inhibit the proliferation of influenza A and B viruses in MDCK cells [27]. Moreover, EGCG, ECG, and EGC were found to prevent the replication of the influenza virus and inhibit neuraminidase activity, with EC_50_ values of 22–28, 22–40, and 309–318 μM, respectively. In vitro experiments confirmed that EGCG and ECG demonstrated hemagglutination inhibition activity and retarded viral RNA synthesis in MDCK cells. However, with the same concentration conditions, EGC did not inhibit RNA synthesis in MDCK cells. [28]. These results suggest that the 3-galloyl group may play a crucial role in antiviral activity against the influenza virus. Recent research has shown that EGCG interferes with sialic acid, which builds on the host cell surface for sialic acid receptors on the viral surface, thus preventing the agglutination reactions induced by influenza and hepatitis viruses [29]. Kim et al. found that EGCG reversed the binding of the virus to the host by lowering the permeability of the viral envelope, thereby impairing the penetration ability of the influenza virus without affecting viral adhesion to the target cell or RNA replication [30]. Tea-derived flavonoids such as quercetin (120 μmol/L) and myricetin (100 μmol/L) had no inhibitory effect on the HA1 subunit of HA but were effective on the HA2 subunit [31]. Zu et al. also confirmed that tea-derived flavonoids could achieve antiviral effects by inhibiting the replication of the *HA* gene of the influenza virus [32].

#### 2.1.2. Neuraminidase (NA)

Neuraminidase (NA) is a glycoprotein spike responsible for cleaving sialic acid from the surfaces of both the viral envelope and host cells, which is essential for hindering viral release and preventing aggregation [31,32]. An experiment utilizing an H5N1 virus detection system and neuraminidase inhibition assay demonstrated that EGCG could specifically inhibit the invasion of H5N1 pseudoviruses and regulate NA activity with IC_50_ values of 145.10 μmol/L and 417.20 μmol/L, respectively. Theaflavins were also found to inhibit NA with an IC_50_ of 129.09 μg/mL [33]. Sahoo et al. further studied the binding site between theaflavin and NA, revealing that theaflavin is capable of establishing hydrogen bonds with specific amino acid residues in NA, including Arg118, Asp151, Asp152, Arg193, Asp199, Asn344, and Arg430, showing a minimum binding energy of 5.21 kcal/mol. Inhibiting NA is beneficial for delaying the release, circulation, and aggregation of progeny viruses [34].

#### 2.1.3. RNA Polymerase

The RNA polymerase of the influenza virus is a heterotrimeric complex including PB1, PB2, and PA subunits. The functionality of this enzyme complex relies on the presence of the 3P proteins [17]. This polymerase is multifunctional, exhibiting not only RNA polymerase activity but also RNA endonuclease activity, which facilitates the degradation of host cell mRNA. Consequently, the endonuclease domain serves as a potential target for anti-influenza A virus therapeutics. The inhibition of endonuclease activity in the PA subunit of the influenza A virus by catechins is primarily due to their galloyl moiety, as demonstrated in various studies [35]. Catechins containing galloyl groups are well-suited to fit into the active site of the endonuclease domain, allowing for stable binding to the influenza virus RNA polymerase [36]. This interaction effectively inhibits the degradation of host mRNA.

#### 2.1.4. Non-Structural Protein 1 (NS1)

The NS1 protein is exclusively present in host cells infected by the influenza virus and represents the sole non-structural protein of the virus [22]. NS1 interacts with host nuclear proteins, inhibiting the export of mRNA from the nucleus. Additionally, NS1 enhances the translation of viral mRNA, thereby promoting viral protein expression while simultaneously suppressing host cell protein synthesis [37]. The NS1 protein exhibits interferon-antagonistic properties primarily due to its interaction with double-stranded RNA (dsRNA). By binding to dsRNA, NS1 impedes antiviral signaling pathways activated by dsRNA, inhibits the synthesis of circulating interferons (IFN-α/β), and prevents the activation of antiviral proteins such as PKR (double-stranded RNA-dependent protein kinase) and 2′-5′ oligoadenylate synthetase (2′-5′ OAS) [38]. In their study, Cho et al. have shown that EGCG effectively inhibited the interaction between NS1 and dsRNA at concentrations below one micromolar [39]. Thermal denaturation experiments revealed that EGCG binds to the dsRNA-binding motif of NS1 containing Arg38. Quinoxaline and its derivatives, which are structurally similar to EGCG, also act as inhibitors by preventing the interaction between NS1 and dsRNA [40].

The anti-flu effects of tea are primarily attributed to catechins, especially EGCG, which exhibit multi-target effects. Further research indicates that the active centers of EGCG’s antiviral activity are located on the phenolic hydroxyl groups and galloyl moieties of the B and D rings [41]. Song et al. demonstrated that catechins with a 3-galloyl group exhibit stronger antiviral activity, with the order of potency being: GCG, EGCG (4 μmol/L) > CG, ECG (64 μmol/L) > GC, EGC (256 μmol/L) > C, EC (8192 μmol/L) [42]. Additionally, EGCG possesses eight hydroxyl groups, which appear to bind to the viral endonuclease domain protein via hydrogen bonds, thereby disrupting RNA replication [43]. Beyond EGCG, the antiviral effects of theaflavins and tea polysaccharides against influenza viruses have also been identified and recognized in recent years, though the related mechanisms remain to be elucidated [26]. Experiments on mice have also shown that EGCG has anti-influenza virus effects. The oral administration of EGCG (40 mg·kg⁻¹·d⁻¹) can reduce the lethality of H1N1 and mitigate the extent of lung tissue damage in mice by reducing the generation of ROS (reactive oxygen species) induced by the H1N1 virus in the host [44]. Lee found that feeding green tea residue at a dose of 100 mg·kg⁻¹·d⁻¹ for five days significantly reduced the viral load in the lungs of mice during the early stages of infection, and this effect was also observed in chickens [45].

While in vitro experiments and some animal studies have indicated that tea consumption or the use of active tea compounds may prevent influenza virus infection, clinical evidence remains inconclusive. Over the past 30 years, research on the epidemiology and clinical applications of tea consumption in relation to influenza has predominantly been conducted in China and Japan, with a focus on three key areas: (1) the relationship between tea consumption, particularly green tea, and influenza virus infection; (2) evaluating the effects of tea via dietary supplement intake; and (3) assessing the efficacy of tea gargling in preventing influenza infection.

Park et al., in an observational study of 2050 children aged 6–14, found that drinking 2–5 cups of green tea daily was significantly negatively associated with influenza infection rates compared to drinking one cup (200 mL) per day, while the consumption of more than 5 cups showed no significant association [46]. In a randomized controlled trial by Rowe et al. involving 108 healthy adults over five months, participants who took green tea capsules (EGCG + theanine, equivalent to approximately 10 cups of green tea) exhibited a 32.1% reduction in influenza symptoms and a 35.6% reduction in symptom duration compared to the control group [47]. Matsumoto’s study demonstrated that the daily intake of capsules containing 378 mg of catechins and 210 mg of theanine lowered the influenza incidence rate from 13.1% in the control group to 4.1% [48]. Furthermore, gargling with black tea extract (0.5%, *w*/*v*) twice daily also helped prevent influenza, whereas green tea extract (0.5%, *w*/*v*) did not have this effect [49]. Thus, larger-scale studies are needed to confirm whether tea can effectively prevent influenza virus infection.

### 2.2. Rotavirus

Rotavirus (RV), a double-stranded RNA virus, was first discovered by scientists in 1973. [50]. Based on its antigenicity, RV can be classified into seven groups. The first three groups can infect both humans and animals, while the remaining four groups infect only animals [51]. Among these, Group A is the primary cause of severe diarrhea in infants [52]; Group B primarily infects adolescents and can cause large outbreaks, but has become controllable without vaccines due to the understanding of its transmission patterns; and Group C circulates among adolescents with a very low incidence. The most common clinical manifestation of rotavirus infection in humans is diarrhea. The pathogenesis mechanism involves RV binding to host epithelial cell surface receptors containing sialic acid residues via VP4, and subsequently binding to the integrin α2β1 subunit via the VP4 DEG sequence to enter the host cell [53]. Following uncoating in the lysosome, the virus develops, assembles, matures, and is released in the cytoplasm. NSP4, a non-structural protein of RV, also acts as an enterotoxin and has been demonstrated to cause diarrhea without intestinal damage [54].

Research on the antiviral properties of tea against rotavirus dates back to 1991 [55]. The inhibitory effects of green tea and tea polyphenols on rotavirus were reported, with the inhibition of the interference of virus adsorption. Subsequently, Gu et al. demonstrated through cellular experiments that, unlike traditional anti-rotavirus (RV) drugs that primarily enhance intestinal immunity, tea extracts can inhibit RV adhesion to the host cell surface, thus mitigating cellular damage [56]. Using transmission electron microscopy (TEM), Lipson found that epigallocatechin gallate (EGCG) and anthocyanins exert a synergistic antiviral effect against rotavirus by disrupting the recognition of host cells and RV [57]. This inhibition primarily occurs by blocking or modifying viral ligands or antigenic epitopes, thereby diminishing the virus’s binding ability [58]. Additionally, theaflavins from black tea have also been shown to possess some anti-rotavirus activity. Clark et al. isolated theaflavins (TF1, TF2A, TF2B, and TF3) from black tea, all of which demonstrated significant inhibitory effects on bovine rotavirus, with TF2A exhibiting the strongest effect, having an EC50 of 251.39 μg/mL. Unlike EGCG, the antiviral efficacy of theaflavins against rotavirus (RV) is primarily attributed to their chelation with proteins [59]. Research has indicated that the activity of Cl^−^ channels on the intestinal cell membrane is closely linked to RV-induced diarrhea. Polypeptides found in black tea can inhibit these Cl^−^ channels by suppressing cyclic nucleotide and Ca^2+^ signaling pathways in intestinal cells, thus alleviating viral diarrhea [60]. Furthermore, researchers have discovered that resveratrol can reverse RV RNA replication [61]. However, similar mechanisms involving catechins or theaflavins have been rarely reported, indicating the need for further experimental validation, and research on the epidemiology of rotavirus and the clinical applications of catechins or theaflavins for rotavirus warrants further attention.

### 2.3. Adenovirus (ADV)

Adenovirus is one of the earliest discovered double-stranded, non-enveloped DNA viruses, exhibiting resistance to human bile and gastric secretions. It commonly replicates in key parts of the human body, such as the pharynx, intestines, conjunctiva, and lymphoid tissues, leading to nervous system disorders, respiratory infections, and inflammation [62]. Adenoviruses are categorized into seven groups (A–G), each of which can affect humans, with a total of 55 identified as pathogenic strains. The most common serotypes are 1–8. The novel serotype 55 is a recombinant virus derived from human adenovirus types 11 and 14, belonging to subgroup B2. The pathogenic mechanism of adenoviruses remains unclear. Researchers hypothesize that the *E1B* gene may produce two proteins, 19 Kd and 55 Kd, which inhibit apoptosis in infected cells. The former directly prevents apoptosis, while the latter binds to the P53 protein in infected cells to exert its inhibitory function [63]. Additionally, the *E1A* gene product can interact with P300 and DPB proteins to inhibit the reactivation of P53 protein [64]. The disruption of interferon protection is another crucial pathogenic mechanism. Finally, adenoviruses can inhibit the expression of receptors by blocking the transcription or mRNA transport of major histocompatibility complex (MHC-1) molecules on the cell surface, interfering with antigen–antibody recognition [65].

In vitro experiments have confirmed that EGCG, EC, ECG, and other compounds exhibit significant inhibitory effects on adenovirus endopeptidase activity, with EGCG showing the lowest IC_50_ value at 109 μmol·L^−1^, while the IC_50_ values for the other compounds range between 245 and 3095 μmol·L^−1^ [66]. Subsequent experiments have demonstrated that epigallocatechin gallate (EGCG) exerts its anti-adenovirus effects both intracellularly and extracellularly. EGCG disrupts the viral protease adenain, thereby reducing viral infectivity and hindering replication within host cells. Additionally, EGCG modulates the expression levels of adenovirus receptor proteins and cysteine proteases, further diminishing the virus’s ability to infect host cells [67]. Ali et al. conducted experiments using HEp2 cells to investigate the effects of black tea extracts on adenovirus infection [68], and it was demonstrated that black tea extracts inhibit adenovirus replication at the post-adsorption stage. Additionally, Liu et al. established a mouse model of adenovirus-induced pulmonary disease, revealing that the activation of the YAP/TAZ-TEAD4 pathway is associated with adenovirus proliferation, migration, and autophagy. However, the relationship between tea functional components and this pathway remains inconclusive [69]. Research on animal experiments and epidemiological investigations of adenovirus still requires further exploration.

### 2.4. Human Immunodeficiency Virus (HIV)

HIV, or human immunodeficiency virus, commonly referred to as the AIDS virus, is a retrovirus responsible for compromising the human immune system [70]. Infected individuals often succumb to complications from infections or cancers. HIV can be classified into two types: HIV-1, originating from chimpanzees, and HIV-2, derived from sooty mangabey monkeys [71]. Current research indicates that HIV targets and depletes CCR5^+^ CD4^+^ T cells by attacking the host immune system. Additionally, HIV can induce the expression of Fas protein, a transmembrane protein, which accelerates the apoptosis of CCR5^+^ CD4^+^ T cells. Due to HIV infection and the depletion of T cells, the body’s immune activation response is triggered, leading to a substantial proliferation of CCR5^+^ CD4^+^ T cells. However, as HIV is not eliminated, this proliferation instead provides a substrate for extensive viral replication. Ultimately, as CCR5^+^ CD4^+^ T cells are extensively destroyed, HIV proliferates, aggregates, and spreads, resulting in severe immune system deficiency, which progresses to Acquired Immune Deficiency Syndrome (AIDS) [72,73,74].

EGCG is the most functional component in tea for preventing HIV infection, due to its structural similarity to non-nucleoside reverse transcriptase inhibitors (NNRTIs). EGCG acts as an inhibitor of HIV reverse transcriptase [75]. The specific mechanism may involve EGCG attaching to the cell surface, causing the deformation of the envelope phospholipids, which promotes their disintegration. The D1 region on CD4^+^ is the binding site for HIV envelope glycoprotein 120. Cell-based studies have shown that at a concentration of 0.2 mmol/L, EGCG is able to prevent glycoprotein 120 from binding to CD4^+^ cells, which is due to its strong affinity for CD4^+^ receptors. Research suggests that theaflavins suppress HIV entry by hindering the fusion process of membranes, facilitated by envelope glycoproteins. This inhibitory action is linked to the prevention of the gp41 six-helix bundle’s assembly, a crucial structure necessary for membrane fusion activity [76,77]. Hauber confirmed that EGCG can inhibit semen-derived HIV [78]. Prostatic acidic phosphatase (PAP) amino acid segment 248-286 (PAP248-286) enhances HIV-1 infectivity and is abundantly secreted in semen, forming β-sheet-rich amyloid fibrils known as semen-derived enhancer of virus infection (SEVI). SEVI captures HIV and facilitates its attachment to target cell surfaces, thereby enhancing receptor-mediated fusion between the virus and host cells. EGCG selectively blocks the function of PAP248-286, breaks down SEVI fibrils, and as a result, hinders HIV transmission [79,80]. Moreover, catechins have been shown to act as apoptosis inducers to prevent HIV infection [81]. A cell-based study on the antiviral effects of Pu-erh tea extracts indicates that Pu-erh ripe tea has a better inhibitory effect compared to raw tea. It can prevent the fusion of normal cells with HIV-infected cells, with an EC_50_ value of 234.27 μg/mL, but it does not inhibit reverse transcriptase activity [82]. Currently, animal experiments and epidemiological investigations on the anti-HIV effects of tea are severely lacking, with research primarily focused on cellular mechanisms.

### 2.5. Human Papillomavirus (HPV)

Human papillomavirus (HPV) is a sexually transmitted, non-enveloped, circular double-stranded DNA virus that targets and infects human epithelial cells [83]. Approximately 100 subtypes of HPV have been reported, with most associated with genital infections. HPV is a pathogen linked to various cancers, such as cervical cancer [84]. In cervical cancer, once HPV enters epithelial cells, it utilizes its *E1* and *E2* genes to complete DNA replication. Subsequently, the *E5*, *E6*, and *E7* genes induce rapid viral proliferation, while the *E4* gene further accelerates DNA replication, enhancing viral propagation [85]. During the late stage of replication, the viral capsid proteins L1 and L2 join together, leading to the formation of new viral particles that are subsequently discharged into the affected epithelial tissue. Viral proteins may facilitate the deregulation of the host’s normal cellular cycle, interfere with key regulatory mechanisms, and modify the signaling environment within the cell, thereby enhancing the conditions that favor viral replication [86]. In differentiated cells, the delayed expression of viral proteins is a primary cause of delayed antigen presentation to host immune cells. Most severe HPV infections are cleared by the host’s immune system after 12–18 months. However, when the immune response is compromised, incomplete viral clearance can lead to persistent infections, resulting in viral accumulation and the potential development of malignant tumors [87,88].

Extensive research suggests that EGCG has a notable preventive effect against cervical cancer caused by HPV infection. In vitro experiments have demonstrated that EGCG can inhibit cervical cancer cells infected with HPV16 and HPV18 subtypes, primarily by targeting cellular telomerase [89]. Besides EGCG, other catechins like EC, EGC, and ECG can also induce apoptosis by disrupting the mitochondrial function of infected cells. Tang et al. indicates that green tea polyphenols induce apoptosis in HPV16 positive cells via the modulation of the Nrf2 pathway, with a dose-dependent effect [90]. Derivatives of polyphenols, including theaflavin, have been demonstrated to reduce the expression of the HPV *E7* gene, which in turn curbs significant viral proliferation [91,92]. Recent findings indicate that EGCG can modulate the ubiquitin-proteasome pathway, promoting the degradation of HPV E6 and E7 proteins [93]. Microtubules and their associated proteins are common targets for anticancer drugs, and studies reveal that EGCG can depolymerize microtubule proteins in infected cells, thereby preventing cancer cell dissemination [92,94,95]. Moreover, EGCG has been shown to block angiogenesis, a process essential for tumor cells to acquire nutrients and oxygen, by reducing the levels of vascular endothelial growth factor (VEGF) and matrix metalloproteinases (MMP-2 and MMP-9) [94,95]. Ahn et al. discovered that the oral administration of green tea extract (containing EGCG and other catechins) led to a reduction in HPV DNA and viral load in approximately 69% of participants [89]. Furthermore, the topical application of the extract proved more effective, achieving a clearance rate of 52–57% for genital warts caused by HPV6 and HPV11 [96,97]. In contrast, the oral administration of EGCG alone (200 mg) did not significantly improve the clearance rate compared to the control group [98]. Consequently, future research should utilize epidemiological methods to further investigate the impact of catechins on cervical cancer.

### 2.6. Hepatitis Virus

Viral hepatitis can be classified into seven types based on the virus type, with hepatitis B and C being the most severe. An estimated 2 billion people worldwide have been infected with hepatitis B and C viruses, and over 350 million people suffer from chronic liver infections [99,100]. Currently, antiviral treatments primarily involve nucleoside analogues and interferon. However, nucleoside drugs only inhibit viral replication without immunomodulatory effects, while interferon drugs present challenges such as dosage difficulties and adverse reactions.

Hepatitis B and C viruses (HBV and HCV) can cause hepatitis, liver cirrhosis, and liver cancer. After entering the body, these viruses not only adhere to the liver cell membrane but also disseminate to other parts of the body through the bloodstream. They simultaneously elicit specific humoral and cellular immune responses [101]. The pathogenic mechanisms of HBV and HCV are not fully understood; however, they may involve the exposure of liver-specific lipoproteins (LSPs). The presence of LSPs can initiate an autoimmune response in liver cells, leading to damage mediated by cytotoxic T lymphocytes (CTLs) or lymphokines [102,103]. Recent research suggests that hepatitis B and C viruses (HBV and HCV) collaboratively accelerate liver fibrosis via a TGF-β1-induced OCT4/Nanog-dependent pathway [104]. Furthermore, the HBsAg antigen secreted by these viruses can form antigen–antibody complexes, potentially causing extrahepatic complications such as glomerulonephritis and arthritis, or accumulating within the liver, resulting in liver tissue necrosis [105].

As early as 2001, researchers confirmed that catechins can reverse liver fibrosis using a duck hepatitis B virus infection model [106]. However, current animal experiments are limited because mice and rats cannot be infected by hepatitis B and C viruses. Consequently, research on the effects of tea on viral hepatitis mainly focuses on in vitro and epidemiological studies. Numerous studies have investigated the inhibitory effects of tea extracts and functional components on hepatitis B and C viruses. It has been reported that green tea extract (1.5%, *w*/*v*) inhibits the expression of HBV-specific antigens and reduces intracellular and extracellular HBV DNA and covalently closed circular DNA (cccDNA) [105]. Subsequently, tea polyphenols from GTEs not only block HBeAg secretion [107], but also down-regulate HBV antigen expression and suppress Farnesoid X receptor α (FXRα) which initiates the transcriptional activation of the EnhII/core promoter [108]. Recent research has indicated that tea polyphenols, especially EGCG, could induce clathrin-dependent Na+-taurocholate transporter (NTCP) endocytosis in the cell membrane to inhibit HBV infection [109,110]. Meanwhile, disrupting the replication of HBV by the ERK1/2-HNF4α axis is also one of the important targets for EGCG [111]. Without green tea, theaflavins in black tea have also been proven to effectively inhibit HCV infection. Theaflavins can act on lysosomes, improving intracellular pH, and reduce the viral DNA replication rate. NS3/4A serine protease is essential for HCV targeted recognition, and theaflavins could further prevent HCV entry into cells by disrupting the structure of NS3/4A serine protease [112]. The HCV cofactor cyclophilin is inhibited by theaflavins to stop HCV replication [113]. The MiR-548m gene is involved in the membrane fusion process between HCV and host cells [114]; theaflavins can block HCV entry into target cells by enhancing miR-548m and preventing HCV attachment [115]. Although animal experiments on hepatitis viruses are relatively scarce, future studies can utilize epidemiological methods to further investigate the antiviral effects of tea components against hepatitis viruses.

### 2.7. Epstein–Barr (EB) Virus

A linear double-stranded DNA virus, Epstein–Barr virus (EBV), encodes around 100 genes, including three significant subtypes: viral capsid antigen (VCA), early antigen (EA), and nuclear antigen (NA) [116,117]. EBV primarily targets B lymphocytes, T lymphocytes, epithelial cells, and natural killer (NK) cells, and it is associated with a range of human diseases, including infectious mononucleosis (IM), chronic active EBV infection (CAEBV), and several EBV-related immunodeficiencies and cancers, such as nasopharyngeal carcinoma, gastric cancer, Burkitt’s lymphoma, Hodgkin’s disease, and T/NK cell lymphoma [118,119]. The pathogenesis of EBV remains unclear. Studies have demonstrated that EBV can trigger autoimmune diseases, resulting in a significant decrease in CD4^+^ cells, a notable increase in CD3^+^ and CD8^+^ cells, and impaired functionality of NK cells and cytotoxic T lymphocytes (CTLs), hindering the timely clearance of EBV-infected lymphocytes [120,121,122]. This leads to the extensive activation and proliferation of EBV-infected lymphocytes, causing the excessive release of cytokines such as IL-13 and subsequent cascade reactions, which activate macrophages and induce macrophage phagocytosis of blood cells, resulting in EBV-associated hemophagocytic lymphohistiocytosis (EBV-HLH) [123]. Furthermore, EBV infection increases tumor risk, primarily via the oncoprotein latent membrane protein 1 (LMP1). One transformation effector site of LMP1, TES2, interacts with the TNF receptor-associated death domain (TRADD) to mediate NF-κB and AP1 activation, thereby inducing B cell proliferation. LMP1 also modulates the expression of the A20 gene, which is linked to the differentiation of nasopharyngeal carcinoma, thus inhibiting the differentiation of nasopharyngeal carcinoma cells. Apoptosis is also associated with LMP1, which can induce the expression of human telomerase reverse transcriptase (HTERT), leading to telomerase activation and promoting cellular transformation [116,124,125].

The induction of apoptosis is a crucial immune mechanism that enables the body to counteract viral invasion. Nuclear factor κB (NF-κB) plays a key role as a regulator of cell growth and is implicated in the oncogenic activity of the Epstein–Barr virus (EBV) in certain malignancies. By binding to the promoter of the epidermal growth factor receptor (EGFR), NF-κB can activate EGFR expression, leading to malignant transformation. Yan et al. demonstrated that epigallocatechin gallate (EGCG) can inhibit LMP1-induced NF-κB activation by modulating the phosphorylation of IκBα, an inhibitor of NF-κB, thereby suppressing the activity of the downstream gene EGFR [126]. In addition, researchers employed pTet-on-LMP1 HNE2 cells to create an LMP1 expression system (tetracycline-inducible expression system). They further discovered that EGCG enhances caspase-9 activity through LMP1, increases the release of mitochondrial cytochrome c, and inhibits Bcl-2 protein expression, thereby suppressing NF-κB expression [127]. Therefore, NF-κB may serve as a potential therapeutic target for Epstein–Barr virus (EBV) treatment.

### 2.8. Coronavirus

In the last thirty years, three coronaviruses capable of human-to-human transmission have been discovered: SARS-CoV (Severe Acute Respiratory Syndrome Coronavirus), MERS-CoV (Middle East Respiratory Syndrome Coronavirus), and SARS-CoV-2 (Severe Acute Respiratory Syndrome Coronavirus 2) [128]. Coronaviruses are RNA enveloped viruses with genomes ranging from 27 to 32 kb, named for the crown-like appearance of their spike proteins [129]. Compared to the SARS outbreak in 2003, COVID-19, caused by SARS-CoV-2, exhibits a lower fatality rate and higher transmissibility [130].

Researchers have demonstrated that SARS-CoV-2 binds to the target cell surface through its spike glycoprotein (S protein) interacting with the angiotensin-converting enzyme 2 (ACE2) receptor. Subsequently, the transmembrane protease serine 2 (TMPRSS2) cleaves ACE2 and activates the S protein, facilitating viral entry and the infection of host cells [131]. ACE2 is expressed in various human tissues, including the lungs, heart, kidneys, testes, and intestines, with particularly high expression in respiratory epithelial cells. Once inside the cell, the virus uncoats to release its RNA, which is then used to produce new virions that are released extracellularly to infect additional cells. Additionally, SARS-CoV-2 can directly induce an excessive immune response that damages lung tissue [132].

Polyphenolic compounds, especially EGCG, have attracted significant attention due to their antiviral properties against coronaviruses. EGCG can bind to the spike protein (S protein) on the surface of the coronavirus, preventing virus attachment to host cell receptors [133]. This mechanism is essential for preventing virus entry into host cells. In addition, EGCG can also improve the fluidity and stability of the cell membrane and further reduce the interaction between the virus and the host cell to enhance its antiviral effect [134]. In addition, EGCG also inhibits the synthesis of coronavirus RNA. The replication of coronavirus in host cells is dependent on the activity of RNA-dependent RNA polymerase (RdRp) [135]. Studies have indicated that EGCG inhibited viral RNA synthesis by interfering with the activity of a polymerase, 3C-like protease (3CLpro), a viral polypeptide processing enzyme. By inhibiting the activity of 3CLpro, EGCG effectively prevents viral maturation and replication, further reducing viral spread within host cells [136,137].

Theaflavins (TFs) demonstrate similar antiviral activities against coronaviruses. Studies have shown that theaflavins which are from black tea are able to change the conformation of viral capsid proteins, thereby preventing the fusion of the virus with the host cell membrane [136]. In addition, theaflavins can also enhance the antiviral response of host cells by inducing the production of interferon and other antiviral factors, thereby inhibiting the replication and spread of viruses [138].

L-theanine, another important component of tea, has also demonstrated potential against coronaviruses. It enhances the activity of natural killer cells and macrophages, which are crucial in the early stages of viral infection [139]. Furthermore, L-theanine promotes antibody production and boosts the host’s antiviral immune response [140].

In addition, caffeine and flavonoids also have an inhibitory effect on coronaviruses. For example, caffeine inhibits phosphodiesterase activity, regulates signal transduction in host cells, and thereby suppresses viral replication [141]. Some flavonoids like myricetin (100 μmol/L) inhibit viral enzymes and interfere with viral RNA synthesis, showing potential antiviral effects against coronaviruses [141]. Compared to coffee, daily tea consumption (5–10g/day) can activate γδT cells in the human body [142]. The precursor of theanine, ethylamine, is a non-peptide molecule that activates γδT cells in the blood, facilitating memory responses upon re-exposure to ethylamine and enhancing immune functions that contribute to resistance against microbial pathogens.

### 2.9. Other Viruses

Porcine reproductive and respiratory syndrome virus (PRRSV), an infectious viral condition, is notable for causing reproductive complications in expectant sows and significant respiratory issues in young piglets [143]. PRRSV predominantly targets alveolar macrophages, including pulmonary intravascular macrophages. Wu et al. established a PRRSV-infected Marc-145 cell model to investigate the inhibitory effects of epigallocatechin gallate (EGCG) on PRRSV. At 48 h post-infection, EGCG exhibited a dose-dependent inhibition of PRRSV adsorption, internalization, and replication at concentrations ranging from 5 to 40 μg/mL. Additionally, palmitoylated EGCG demonstrated approximately 24.3% higher bioavailability compared to EGCG. Compared to EGCG, which had CC_50_ and EC_50_ values of 2359.71 μM and 8.53 μM respectively, the observed half-maximal cytotoxic concentration (CC_50_) of 431.42 μM and 50% effective concentration (EC_50_) of 0.48 μM were notably lower [144]. Similar findings have been corroborated by other researchers [145]. These results suggest that converting EGCG to EGCGP enhances its efficacy, providing a novel approach for the development of anti-PRRSV therapeutics.

The herpes simplex virus (HSV), an enveloped DNA virus, is categorized into three distinct types: α, β, and γ. It primarily affects the host’s skin and nervous tissues and is among the oldest known human viruses. HSV establishes latency in neurons after infecting peripheral tissues, thereby evading the host’s immune response. The virus reactivates to cause recurrent infections when the host’s immune system is compromised [146]. Research has shown that epigallocatechin gallate (EGCG) from green tea effectively inhibits HSV activity under both acidic and alkaline conditions, with the strongest inhibition observed at pH 5.7 [147]. The glycan receptors on the HSV surface and the envelope glycoproteins are among the targets of catechins, which prevent the virus from binding to the host cells [148,149].

Several studies have also reported the inhibitory effects of tea and its functional components on various viruses [98,133,145,146]. For instance, epigallocatechin gallate (EGCG) can interact with the envelope of arboviruses, impeding viral replication [150]. Additionally, tea exhibits efficacy against other viruses, such as Coxsackievirus B, Ebola virus, and hematopoietic necrosis virus [98,133,151].

## 3. Conclusions and Future Perspectives

We have summarized the antiviral mechanisms of different functional components of tea in Table 1. As mentioned, the majority of studies conclude that these mechanisms primarily involve hindering the virus’s ability to attach to receptors on host cells, impeding viral replication, interfering with signal transduction pathways involved in viral proliferation (such as tyrosine kinases), reducing viral reverse transcriptase activity, and enhancing host immunity. Most of these preventive mechanisms focus on the viral infection period.

Most antiviral research currently focuses on EGCG, primarily in green tea. This is closely related to the high content of EGCG in green tea. Studies show that among the six major types of tea, green tea has the highest polyphenol content, while black tea has the lowest, with a difference of 1.8 times between the two teas. However, EGCG did not differ in function and structure between green and black tea [152]. Further research is needed to determine whether, in addition to green and black tea, other varieties such as yellow, dark, oolong, and white tea also exhibit inhibitory effects on post-infection complications, including virus-induced inflammation.

Despite promising in vitro and in vivo studies, much of the current research has been conducted using cellular and animal models. There is a notable scarcity of epidemiological studies that could validate these findings in human populations and the relationship between viral load and daily tea consumption remains unclear. Therefore, the functional components of tea are frequently used as a therapeutic supplement for antiviral treatment. The antiviral mechanisms of tea components are still largely speculative, necessitating further investigation to elucidate their interactions at the molecular level. Future research should aim to explore the full spectrum of tea’s antiviral properties across different virus families and expand beyond catechins and theaflavins to include other bioactive compounds present in various tea types.

## Figures and Tables

**Table 1:** Antiviral activity mechanisms of individual tea components.

Viruses	Tea or Tea Compounds	Mechanisms	References
Influenza virus	EGCG and theaflavins	Regulation of eight viral gene segments	[20,26,28,31,32]
		Inhibition of viral entry	[30,38]
	EGCG	Disruption of viral replication	[43]
		Reduction of ROS	[44]
	Flavonoids	Inhibiting the replication of the *HA* gene	[31]
Rotavirus	Catechins	Interference of virus adsorption	[55]
	EGCG	Arrest of viral and host recognition	[57,58]
	Theaflavins	Chelation with virus proteins	[59]
	Black tea	Inhibition of Cl− channel in host cells	[60]
		Disruption of RV RNA replication	[61]
Adenovirus	Catechins	Disruption of the viral protease adenain	[66,67]
	Theaflavins	Disruption of viral replication	[68]
HIV	EGCG	An inhibitor of HIV reverse transcriptase	[75]
		Reduction of viral activity in sperm	[76]
		An inhibitor of Prostatic acidic phosphatase (PAP) amino acid segment 248–286	[78]
	Pu-erh tea extracts	Avoid host infection with HIV	[82]
	Theaflavins	Modulation of cell membrane properties	[76,77]
		Inhibition of viral entry	
HPV	EGCG	Inhibition of viral entry	[85]
		Reducing the expression of the HPV *E7* gene	[91,92]
		Degradation of HPV *E6* and *E7* proteins	[93]
		Reduction of the levels of VEGF, MMP-2, and MMP-9	[94,95]
	Catechins	Modulation of the Nrf2 pathway	[90]
Hepatitis virus	Green tea extract	Inhibition of the expression of HBV-specific antigens and HBV DNA	[105]
	Tea polyphenols	Down-regulate HBV antigen expression and suppress Farnesoid X receptor α	[108]
		Inducing clathrin-dependent Na+-taurocholate transporter (NTCP) endocytosis in the cell membrane	[109,110]
		Reduction of viral DNA replication	[111]
	Theaflavins	Stopping HCV replication	[113]
Epstein–Barr (EB) virus	EGCG	Modulating the phosphorylation of EB-IκBα	[126]
		Suppressing the expression of NF-κB	[127]
Coronavirus	EGCG	Preventing virus attachment to host cell receptors by binding to the spike protein	[134]
		Improving the fluidity and stability of the cell membrane	[135]
		Inhibition of the synthesis of coronavirus RNA	[136]
		Inhibiting the activity of 3CLpro	[137]
	Theaflavins	Changing the conformation of viral capsid proteins	[137]
		Enhancing the antiviral response of host cells	[139]
	Theanine	Enhancing the activity of natural killer cells and macrophages	[140]
		Boosting the antiviral immune response of the host	[141]
	Caffeine	Inhibiting phosphodiesterase activity, regulating signal transduction in host cells, and suppressing viral replication	[142]
	Flavonoids	Inhibiting viral enzymes and interfering with viral RNA synthesis	[143]
PRRS	EGCG	Inhibition of PRRSV adsorption, internalization, and replication	[144]
HSV	EGCG	Inhibiting HSV activity under both acidic and alkaline conditions	[146,147]
		Preventing the virus from binding to the host cells	[148,149]
Arboviruses	EGCG	Impeding viral replication	[150]
